# Translation and cross-cultural adaptation of the Toronto extremity salvage score system into Arabic and its validity

**DOI:** 10.1186/s13018-025-05980-0

**Published:** 2025-07-28

**Authors:** Ibrahim Alshaygy, Musab Alageel, Abdulrahman Alaseem, Motaz Alogayyel, Anthony Griffin, Mahmoud Shaheen, Abdulaziz Alsudairi, Khalid Murrad, Sarah Alqahtani, Rana Alqahtani, Furqan Alawami, Nizar Algarni, Waleed Albishi, Fawzi Aljassir

**Affiliations:** 1 Department of Orthopedic Surgery, College of Medicine, King Saud University, Riyadh, Saudi Arabia; 2 Musculoskeletal Oncology Unit, Orthopaedic Surgery, Mount Sinai Hospital, Toronto, Canada; 3 King Faisal Specialist Hospital & Research Centre (KFSH-RC), Riyadh, Saudi Arabia; 4 College of Medicine, Almaarfa University, Riyadh, Saudi Arabia

**Keywords:** Validation, Cross- cultural adaptation, TESS, Tumor, Arabic Language

## Abstract

**Background:**

The treatment of musculoskeletal (MSK) tumors involving the extremities has evolved over the past decade with the introduction of prosthesis and new chemotherapy regimen. The Toronto Extremity Salvage Score system (TESS) is a patient-filled questionnaire that measures the functional status of patients with MSK tumors who underwent limb-salvaging procedure. The purpose of this study is to translate TESS into Arabic (TESS-AR) and to examine its reliability and validity.

**Methods:**

Our study is a multi-center in Riyadh, Saudi Arabia. Arabic-speaking adults diagnosed with MSK tumors involving the extremities were included. TESS-AR was created following clear, user-friendly guidelines for translation. Moreover, reliability and validity were measured using the test-retest method and construct validity, respectively.

**Results:**

108 participants completed the TESS-AR, 56% had lower limb tumors. The participants reported that the TESS-AR was clear and all questions and answers were understood. The test-retest reliability showed excellent reliability, with an interclass correlation coefficient of 0.965 for both the lower and upper extremity TESS-AR. Cronbach’s alpha of lower extremity TESS-AR was 0.972, whereas that of upper extremity TESS-AR was 0.969, indicating strong internal consistency. The construct validity between TESS-AR and SF-36 showed a strong and moderated correlation between most of the components, with a Pearson correlation coefficient >0.40. Similar results were found between TESS-AR and EORTC QLQ C30.

**Conclusion:**

The TESS-AR is a comprehensible, valid, and reliable score for assessing functional outcomes in patients with extremity tumors. We believe that TESS-AR can be used by clinicians, researchers, and patients alike.

## Background

Until the 1980s, amputation of the limb was the main surgical treatment for extremity sarcomas [7, 16]. The advent of improved cross-sectional imaging, such as MRI, reconstructive options, such as endoprostheses and bulk allografts for large bone defects, and the use of neoadjuvant chemotherapy and radiation therapy permitted a switch from primary amputation to limb salvage surgery in many cases. Several studies have shown that moving from primary amputation to limb salvage could be done without compromising local tumor control [2–11]. Evolving reconstructive options has led to improvements in patient function and quality of life. Simultaneously, the items to measure and report on patient outcomes have also evolved. For sarcoma patients, assessments such as the initial Musculoskeletal Tumor Society Score scale and its revised version were physician completed [8]. Generic health status measuring tools, such as Short Form-36 (SF-36), were developed more for community-based, independently living populations [4, 6], while more cancer-specific measures, such as EORTC-QLO-C30, do not assess physical function adequately [6].

TESS was developed as a disease-specific measure of functional outcomes for patients undergoing limb-sparing surgery for musculoskeletal tumors of the extremities [6]. Its content was developed in conjunction with sarcoma patients and has been found to be valid, reliable, and representative [5, 6]. There is both a 30-item lower extremity questionnaire and a 29-item upper extremity questionnaire measuring functional difficulties in performing activities of daily living. Questions are rated on a 5-point Likert scale from ‘Not at all difficult’ to ‘Impossible to do’, and a score out of 100 is calculated. This permits comparisons of functional outcomes for different surgical procedures from a patient perspective. It has international acceptance, and while developed in English, it has been translated into multiple other languages, including Japanese, Chinese, Dutch, Danish, Finnish, and Korean [1, 9, 10, 12, 15, 17]. An Arabic version has not yet been translated and validated.

### Questions/purposes

This study aimed to translate TESS into Arabic, perform cross-cultural adaptation, and examine the reliability and validity of the Arabic version with the help of patients who underwent limb-salvage procedures for sarcoma. Furthermore, the correlation between Arabic TESS with SF-36 and EORTC quality of life C30 was also evaluated.

## Material and methods

### Study design and setting

Our study was conducted in the orthopedic out-patient clinics at King Saud University Medical City (KSUMC) and King Faisal Specialist Hospital and Research Center (KFSH-RC) between February 2020 and June 2020. The patients eligible for the study included adults aged 18 and above who could speak, read, and write Arabic and who had been diagnosed with a tumor of the upper or lower extremity. Additionally, it includes either preoperative or postoperative surgery. The translation was undertaken first, followed by testing for reliability and cross-cultural adaptation.

#### Translation

**Fig. 1:**
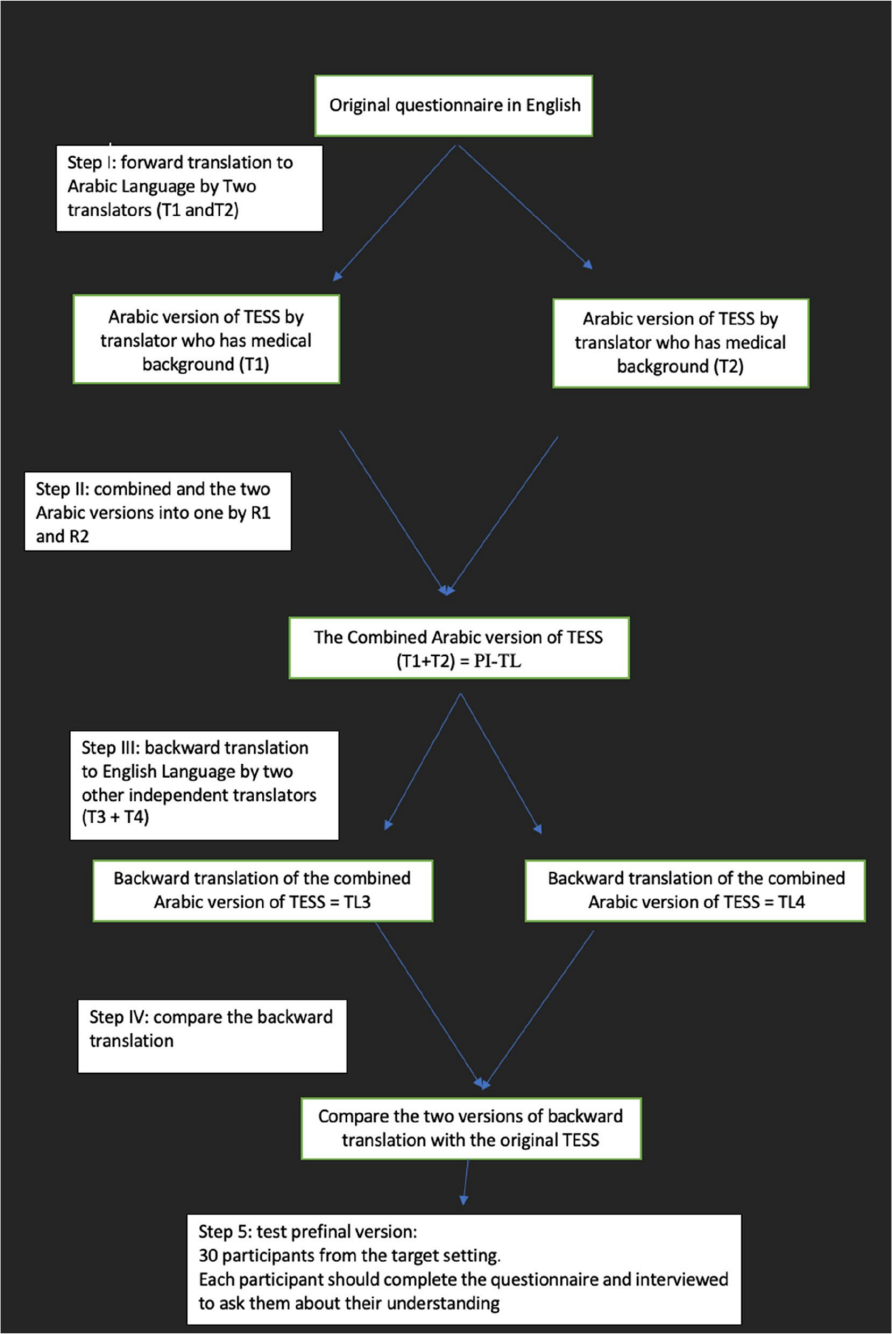
Translation process of the TESS questionnaire into Arabic

We initially started with a forward translation of the TESS into Arabic, followed by a back translation into English. The scale was translated into Arabic by two independent translators whose primary language is Arabic [13]. Both translators are fluent and well experienced in the cultures of the two languages. The first translator has a background in medical terminology and experience in clinical orthopedics and is knowledgeable about the construct of the instrument. The second translator did not have a medical background and no previous experience with the construct of the instrument. The translated version from the first translator was labeled TL1, and the second version of the second translator was labeled TL2. The translated and original versions of TESS were compared by a third party, who is bilingual and bicultural. No significant difference was observed between the two. Following group consensus, we adopted one final version, labeled PI-TL. The scale was then translated back from the Arabic version into English by two other translators whose mother tongue is English. The English translators have extensive experience in translating medical studies. Then we compared the two back translations with each other and compared them to the original instrument, and no differences were found. The final Arabic version of TESS (TESS-AR) was produced (Fig. 1).

A pilot study was conducted involving 30 participants to determine if there was any difficulty in understanding the contents of the scale. The participants stated that the TESS-AR (upper and lower) questionnaire was clear and comprehensive.

#### Patient selection

Patients who had tumors involving the upper or lower extremities and had limb-salvage procedures in KSUMC and KFSH-RC consented to participate in our study. The patients were separated into two groups: the first group consisted of those diagnosed with upper extremity tumors, and the second group consisted of those diagnosed with a lower extremity tumor. The first group completed the upper extremity TESS-AR, while the second group filled the lower extremity TESS-AR.

#### Reliability and validity

To determine the reliability, we used test-retest reliability. Each group answered the questionnaire twice in two different periods of around 14 days. Initially, each patient completed only the appropriate TESS-AR. During the second period, the participants answered two questionnaires TESS-AR (upper or lower) and either SF-36 or EORTC-QLQC30 to determine construct validity.

### Ethical consideration

Approval from the Institutional Review Board (IRB) in the Department of Family and Community Medicine in the College of Medicine, King Saud University and the IRB in King Faisal Specialist Hospital & Research Centre was obtained prior to commencing the study.

The participants signed their consent after the purpose of the study was explained to them, and they understood they had the right to withdraw at any time without any obligation to the study team. All questionnaire data were collected with a study number, and no identifying information was included on the questionnaire to ensure anonymity. No incentives or rewards were given to the participants.

### Statistical analysis

Data were analyzed using the Statistical Package for Social Studies (SPSS 22; IBM Corp., New York, NY, USA). Continuous variables were expressed as mean ± standard deviation, and categorical variables were expressed as percentages.

The Pearson correlation coefficient was used to assess the correlation between the TESS and and SF-36. Cronbach’s alpha was used to assess the reliability and internal consistency (ICC) of the items in the TESS questionnaire. A *p*-value <0.05 was considered statistically significant.

## Results

A total of 108 participants diagnosed with a tumor of the upper or lower extremities were included in the study, out of which 12 patients were pre-operative. A total of 56 participants who were diagnosed with lower limb tumors completed the lower extremity TESS-AR questionnaire, and 42 participants who were diagnosed with upper limb tumors completed the upper extremity TESS-AR questionnaire.

Based on the participants’ feedback, both the upper and lower extremity TESS-AR questionnaires were clear and comprehensible.

### Reliability

As shown in Table 1, the mean scores of the lower TESS and upper TESS questionnaires were 78.4 (range 7.3–100) and 84.9 (range 25–100), respectively. Test-retest reliability showed excellent reliability with an interclass correlation coefficient of 0.965 for both the lower and upper extremity TESS-AR questionnaires. Additionally, Cronbach’s alpha of the lower extremity TESS-AR questionnaire was 0.972, whereas Cronbach’s alpha of the upper extremity TESS questionnaire was 0.969, indicating strong internal consistency.

**Table 1:** Test–retest data for the total score of the Japanese version of the TESS

		TESS score^a^				ICC^b^	95% CI		Cronbach’s Alpha^c^
		Min	Max	Mean	SD		Lower	Upper	
Lower	Test	7.26	100.00	78.39	19.26	.965^c^	.931	.987	.972
	Retest	77.50	99.24	92.24	6.96				
Upper	Test	25.00	100.00	84.94	17.39	.965^c^	.928	.988	.969
	Retest	78.57	100.00	93.17	5.50				

*SD* standard deviation, *ICC* infraclass correlation coefficient, *CI* confidence interval

^a^The total score of 100 points, best score; 0, worst score

^b^The ICC ranges from 0.00 (no agreement) to 1.00 (perfect agreement) and describes the consistency of repeated assessments when no real change has occurred for a subject within the assessment period

^c^This estimate can vary between 0.00 (no correlation) and 1.00 (perfect correlation), where a good correlation (e.g., alpha >0.90) among the items indicates strong internal consistency

### Validity

Two other generic questionnaires were employed to perform the construct validity test of the Arabic version of TESS with the SF-36 and EORTC QLQ C30.

### Constructive validity of TESS with SF-36

#### Lower extremity TESS AR

The SF-36 questionnaire was given to 21 participants who answered both the upper and lower limb TESS-AR questionnaire, the results of which are summarized in Table 2. The correlations between the TESS-AR questionnaire and physical functioning, role limitations due to physical health, and general health sections of SF-36 were 0.664, 0.607, and 0.670, respectively, which indicates a strong correlation. Correlations between lower limb TESS-AR and role limitations due to emotional problems, energy fatigue, social functioning, and pain sections of SF-36 results were 0.541, 0.516, 0.553, and 0.414, respectively, which indicates moderate correlation. However, the correlation between lower limb TESS-AR and emotional well-being was 0.261, which is a weak correlation.

**Table 2:** Criterion validity data for the TESS (Lower extremity) as compared with SF - 36

SF-36 components	Mean	SD	Pearson correlation coefficient^a^	95 % CI		*P* value
				Lower	Upper	
Physical functioning	51.80	19.41	0.664	0.341	0.987	<0.001
Rolelimitations due to physical health	58.00	41.91	0.607	0.264	0.950	0.001
Role limitations due to emotional problems	74.67	37.61	0.541	0.179	0.904	0.005
Energy fatigue	65.20	22.43	0.516	0.147	0.886	0.008
Emotional well being	74.72	17.19	0.261	−0.155	0.678	0.207
Social functioning	67.80	27.68	0.553	0.194	0.913	0.004
Pain	77.00	23.42	0.414	0.021	0.806	0.040
General health	67.00	19.20	0.670	0.349	0.990	<0.001

^a^ The strength of the correlation was categorized in accordance with the absolute value of the correlation coefficient as follows: <0.20 (very weak); 0.20–0.39 (weak); 0.40–0.59 (moderate); and ≥0.60 (strong)

#### Upper extremity TESS AR

A total of 19 patients completed both the upper limb TESS-AR and SF-36 questionnaires to assess the correlation, the results of which are summarized in Table 3.

**Table 3:** Criterion validity data for the TESS (Upper extremity) as compared with SF - 36

SF-36 components	Mean	SD	Pearson correlation coefficient^a^	95 % CI		*P* value
				Lower	Upper	
Physical functioning	55.00	21.98	0.896	0.385	1.407	0.006
Rolelimitations due to physical health	67.86	47.25	0.824	0.174	1.475	0.023
Role limitations due to emotional problems	90.48	16.27	−0.046	−1.194	1.103	0.923
Energy fatigue	45.71	7.87	−0.114	−1.256	1.028	0.808
Emotional well being	27.43	26.68	−0.727	−1.516	0.063	0.064
Social functioning	68.93	13.83	0.659	−0.205	1.524	0.107
Pain	58.57	11.07	0.399	−0.655	1.453	0.376
General health	78.57	13.14	0.185	−0.944	1.315	0.691

^a^The strength of the correlation was categorized in accordance with the absolute value of the correlation coefficient as follows: <0.20 (very weak); 0.20–0.39 (weak); 0.40–0.59 (moderate); and ≥0.60 (strong)

The correlations between the TESS-AR questionnaire and the physical functioning, role limitations due to physical health, emotional well-being, and social functioning sections of SF-36 were 0.896, 0.824, 0.727, and 0.659, respectively, which indicates a strong correlation. The correlation between the lower limb TESS-AR and the pain section of SF-36 was 0.4, which indicates a moderate correlation. The correlations between the lower limb TESS-AR and the general health, role limitations due to emotional problems, and energy fatigue sections of SF-36 were 0.185, 0.046, and 0.114, respectively, which indicates a very weak correlation.

### Construct validity with EORTC QLQ C30

#### Lower extremity questionnaire

Among the participants who were diagnosed with lower limb tumors, 26 patients answered both the lower limb TESS-AR and EORTC QLQ C30 to assess construct validity. The correlation between lower limb TESS-AR and the social functioning section of EORTC QLQ C30 was 0.605, which indicates a strong correlation. The correlation of the lower limb TESS-AR and the pain, role functioning, and physical functioning sections of EORTC QLQ C30 was 0.588, 0.563, and 0.450, respectively, which indicates moderate correlation. A weak correlation was found between the lower limb TESS-AR and the emotional and cognitive functioning sections of the EORTC QLQ C30, where the Pearson correlation coefficients (PCC) were 0.314 and 0.255, respectively. Lastly, a very weak correlation of 0.087 was found between lower limb TESS-AR and the fatigue section of EORTC QLQ C30. The results of the correlation between the lower limb TESS-AR and the EORTC QLQ C30 are summarized in Table 4.

**Table 4:** Criterion validity data for the TESS (Lower extremity) as compared with EORTC QLQ-C30

EORTC QLQ-C30 components	Mean	SD	Pearson correlation coefficient ^a^	95 % CI		*P* value
				Lower	Upper	
score Physical functioning	14.58	15.44	−0.450	−0.987	0.085	0.092
score Role functioning	12.50	27.55	-.563-^a^	−1.087	−0.070	0.029
score Emotional functioning	90.10	10.19	0.314	−0.260	0.899	0.255
score Cognitive functioning	87.50	23.96	0.255	−0.326	0.840	0.359
score Social functionin	92.71	21.92	.605^a^	0.131	1.115	0.017
score Fatigue	17.36	14.04	−0.087	−0.669	0.498	0.757
score pain	20.83	24.72	-.588-^a^	−1.082	−0.105	0.021

^a^ The strength of the correlation was categorized in accordance with the absolute value of the correlation coefficient as follows: <0.20 (very weak); 0.20–0.39 (weak); 0.40–0.59 (moderate); and ≥0.60 (strong)

#### Upper extremity questionnaire

Seven patients with upper limb tumors answered both the upper limb TESS-AR and EORTC QLQ C30 questionnaires. A strong correlation of 0.613 was found between the upper limb TESS-AR and the role functioning section of EORTC QLQ C30. Moderate correlations of 0.593, 0.498, 0.440, and 0.427, respectively, were found between upper limb TESS-AR and the emotional functioning, pain, social functioning, and physical functioning sections of EORTC QLQ-C30. These results are summarized in Table 5.

**Table 5:** Criterion validity data for the TESS (Upper extremity) as compared with EORTC QLQ-C30

EORTC QLQ-C30 components	Mean	SD	Pearson correlation coefficient^a^	95 % CI		P value
				Lower	Upper	
score Physical functioning	17.46	14.53	−0.427	−0.937	0.054	0.077
score Role functioning	19.05	26.50	-.613-^**^	−1.067	−0.200	0.007
score Emotional functioning	84.92	18.56	.593^**^	0.175	1.075	0.009
score Cognitive functioning	94.44	10.97	−0.040	−0.593	0.510	0.876
score Social functionin	91.27	17.17	0.440	−0.038	0.971	0.067
score Fatigue	29.10	27.55	−0.097	−0.646	0.445	0.702
score Nausea and vomiting	6.35	19.35	−0.280	−0.847	0.246	0.261
score pain	33.33	32.91	-.498-^a^	−0.977	−0.039	0.036

^a^The strength of the correlation was categorized in accordance with the absolute value of the correlation coefficient as follows: <0.20 (very weak); 0.20–0.39 (weak); 0.40–0.59 (moderate); and ≥0.60 (strong)

**Correlation is significant at the 0.01 level (2-tailed)

## Discussion

The TESS questionnaire is currently most widely used to evaluate patients with upper or lower limb tumors. However, no Arabic version has been validated.

In the translation process and the cross-cultural adaptation, there were no difficulties in translating and adapting the items and answers of the upper and lower limb TESS questionnaire into Arabic. Thus, the Arabic questionnaire was comprehensibly based on the participants’ feedback. This is similar to what was found in other adaptation studies [9].

A test-retest method was used to assess the reliability of TESS-AR. For the lower limb TESS-AR, the intra-class correlation coefficient was 0.965, with a Cronbach’s alpha of 0.972, which showed excellent reliability. This is comparable to the original TESS questionnaire [6]. This finding was also seen in many other studies, including a Japanese study that assessed the validity and reliability of TESS in Japanese, where the ICC was 0.941 with a Cronbach’s alpha of 0.972, which is considered excellent reliability [1]. Furthermore, similar results were seen in the Korean, Danish, Chinese, and Dutch studies, where the ICC were 0.874, 0.88, 0.893, and 0.963, respectively, and their Cronbach’s alpha was 0.978, 0.94, 0.953, and 0.957, respectively [1, 10, 12, 14] when looking at the upper limb sections of TESS-AR. This study revealed an ICC of 0.965 with a Cronbach’s alpha of 0.969, indicating excellent reliability. Similar results were also identified in the original TESS and in many other studies. The ICC found in the Japanese, Korean, Danish, Chinese, and Dutch studies was 0.941, 0.979, 0.96, 0.932, and 0.969, respectively, whereas their Cronbach’s alpha was 0.978, 0.989, 0.90, 0.921, and 0.938, respectively, which is considered excellent reliability in all the mentioned studies [1, 9, 10, 14, 15].

Since TESS-AR is a patient-completed questionnaire, we measured construct validity with 2 other patient-reported questionnaires—the SF-36 and EORTC QLQ C30 were used to assess the correlation with the TESS-AR. Other studies utilized these questionnaires to assess the correlation: the Japanese and Dutch groups used SF-36 [1, 15], while the Chinese and Danish groups used the EORTC QLQ C30 to assess the correlation [12, 14].

In the current study, we found a strong correlation between lower limb TESS-AR and physical functioning, role limitations due to physical health, and general health sections of SF-36. Compared to our findings, the Dutch study found a strong correlation between lower limb TESS and both the physical functioning and role limitation sections of SF-36 [10].

However, the correlation with the general health section was moderate [10]. The Japanese study revealed a strong correlation between the lower extremity TESS questionnaire and both physical functioning and role limitation due to the physical health of SF-36 but a weak correlation with the general health section [1]. We found a moderate correlation between role limitations due to emotional problems, energy fatigue, social functioning, and pain and the lower limb TESS-AR questionnaire. The Dutch study, on the other hand, found a moderate correlation with role limitations and emotional problems sections and a strong correlation between the energy, pain, and social functioning sections of SF-36 [10]. The Japanese study found a moderate correlation between pain, energy, social functioning, and emotional problems sections of SF-36 with the lower limb TESS questionnaire [1]. Lastly, we found a weak correlation between emotional well-being and the lower limb TESS-AR questionnaire. However, the Dutch study found a moderate correlation between emotional well-being and the lower limb TESS questionnaire [10]. On the other hand, the Japanese study found a very weak correlation between emotional well-being and lower limb TESS [1].

This study revealed a strong correlation between the upper limb TESS-AR questionnaire and the physical functioning, role limitations due to physical health, emotional well-being, and social functioning sections of SF-36. Similar results are seen in the Dutch study in regards to physical functioning and role limitations due to physical health sections of SF-36. On the other hand, social functioning and emotional well-being were moderately correlated with the upper limb TESS questionnaire [9]. While the Japanese study matches our finding for physical functioning and role limitations due to physical health sections, a moderate correlation was seen between social functioning and upper limb TESS and a very weak correlation between emotional well-being and upper limb TESS. Also, a moderate correlation was seen between upper limb TESS-AR and the pain sections of SF-36. On the other hand, the Dutch study found a strong correlation, and the Japanese study found a weak correlation [1, 10]. Finally, we found a very weak correlation between the upper limb TESS-AR and the general health, energy fatigue, and role limitations due to emotional problems sections of SF-36, while the Dutch study found a moderate correlation of the mentioned sections [9]. However, the Japanese study found a moderate correlation with the energy fatigue and role limitations due to emotional problems sections of SF-36 and a weak correlation with the general health section [1].

We found a strong correlation between lower limb TESS-AR and the social functioning section of the EORTC QLQ C30 with a PCC of 0.605, while a moderate correlation was found in the physical function and role functioning sections with PCC of 0.450, and 0.563, respectively. The Chinese study, however, found a strong correlation between lower limb TESS and the physical function, role functioning, and social functioning sections of the EORTC QLQ C30, with PCC of 0.782, 0.638, and 0.712, respectively [17]. Our study has a few limitations. First, some of the participants had some trouble recalling some information due to the long gap between the first and second questionnaires. Second, the questionnaire was given to participants with different treatment plans, so it may affect the outcomes.

TESS-AR is a comprehensible, valid, and reliable method to assess functional outcomes in patients with extremity tumors. Therefore, we believe that the TESS-AR questionnaire can be used by clinicians, researchers, and patients to report on functional outcomes for patients treated for extremity tumors.

## Data Availability

No datasets were generated or analysed during the current study.
